# Using Cross-Cultural Consumer Liking Data to Explore Acceptability of PGI Bread—Waterford Blaa

**DOI:** 10.3390/foods9091214

**Published:** 2020-09-01

**Authors:** Rachel Kelly, Tracey Hollowood, Anne Hasted, Nikos Pagidas, Anne Markey, Amalia G. M. Scannell

**Affiliations:** 1 UCD School of Agriculture and Food Science, University College Dublin, Belfield, Dublin D04 V1W8, Ireland; rachel.kelly.3@ucdconnect.ie (R.K.); anne.markey@ucd.ie (A.M.); 2 Sensory Food Network, Dublin D15 DY05, Ireland; 3 Sensory Dimensions, Unit F1-F2, Cowlairs, Nottingham, Nottinghamshire NG5 9RA, UK; tracey@hollowood.myzen.co.uk; 4 QI Statistics, Penhales, Ruscombe Lane, Ruscombe, Reading RG10 9JN, UK; anne@qistatistics.co.uk; 5 Kerry Europe & Russia, Millennium Park, Naas, Co., Kildare W91 W923, Ireland; nikos.pagidas@kerry.com; 6 UCD Institute of Food and Health, University College Dublin, Belfield, Dublin D04 V1W8, Ireland

**Keywords:** Waterford Blaa, cross-cultural consumer differences, consumer liking, sensory attributes, gender differences, age differences, PGI status

## Abstract

Waterford Blaa is one of only four Irish food products granted protected geographical (PGI) status by the European Commission. This study aimed to determine whether cultural background/product familiarity, gender, and/or age impacted consumer liking of three Waterford Blaa products and explored product acceptability between product-familiar and product-unfamiliar consumer cohorts in Ireland and the UK, respectively. Familiarity with Blaa impacted consumer liking, particularly with respect to characteristic flour dusting, which is a unique property of Waterford Blaa. UK consumers felt that all Blaas had too much flour. Blaa A had the heaviest amount of flouring and was the least preferred for UK consumers, who liked it significantly less than Irish consumers (*p* < 0.05). Flavour was also important for UK consumers. Blaa C delivered a stronger oven baked odour/flavour compared to Blaa A and was the most preferred by UK consumers. Irish consumer liking was more influenced by the harder texture of Blaa B, which was their least preferred product. Age and gender did not impact liking for Blaas within Irish consumers, but gender differences were observed among UK consumers, males liking the appearance significantly more than females. This is the first paper comparing Waterford Blaa liking of naïve UK consumers with Irish consumers familiar with the product.

## 1. Introduction

In Ireland, bread and bread products markets were estimated to be worth €605 million in 2018, with 29% of Republic of Ireland consumers buying bread rolls/baps from traditional artisan bakeries. This was the second most popular type of bread purchased from these bakeries after baguettes/bagels [1]. Waterford Blaa is a white bread product produced specifically in traditional bakeries in county Waterford and a region of south Kilkenny in Ireland. Waterford Blaa obtained its Protected Geographical Indication (PGI) status in 2013 and is one of only four food products in Ireland with this status [2].

Age, gender, and cultural background have been studied in relation to consumer liking of numerous food and beverage products. Older consumers rated wines higher than middle aged and young consumers for evoking positive emotions, e.g., active, enthusiastic, and good natured [3]. Older consumers also liked jam-filled cakes more [4] and rated berries higher for usage, familiarity, and liking than younger participants [5]. The impact of gender on sensory perception has also been studied by a number of authors [6,7,8], and product dependent gender differences [3,5] have been associated with several food products, including spicy food [9] and wine [3,10].

Cross-cultural differences have been seen when comparing the perceptions of populations from different ethnic backgrounds. Japanese consumers were found to be more tolerant of changes in taste intensity of orange juice and grapefruit juice than Australian consumers [11], Norwegian consumers liked whole grain breads more than consumers from Estonia, Scotland, and Czech Republic [12], and more recently, the reported sensory perception of Balsamic vinegar among product–familiar consumers in Italy was different to those reported by Korean consumers unfamiliar with the product [13]. Geographically close, the populations of the UK and Ireland have been shown to be quite similar in terms of consumer behaviour. In a cross-cultural study [14], results from the Food Related Lifestyle (FRL) survey found that UK and Irish consumers had identical response styles. That said, there is anecdotal evidence of differences in food liking between the UK and Ireland, e.g., the Irish palate tends to prefer saltier potato chips (crisps) and sweeter breakfast cereal, however, published research comparing the food preferences of Irish and UK consumers is limited. 

Cross-cultural differences in opinions of pre-sliced white and brown bread have previously been studied comparing Chinese-Malaysian and Australian consumers [15]. Authors found that, whilst both cultures were affected by product information, Australian consumers were more receptive to information on fibre content than the Chinese-Malaysian consumers. The impact of age on consumer perception of rye bread [16] determined that younger and older Swedish consumers differed in their liking of rye bread but held similar perceptions of its healthiness. Age (18–44 vs. 45–80 years) and gender were also found to impact the selection of descriptive attributes and perceived health effects of bread, and consumers with a lower education level struggled to identify healthy bread compared to more educated Swedish consumers [17]. 

Liking of food as informed by consumer perception of its sensory attributes has a considerable impact on food choice and self-reported purchase intent [18]. There are, of course, many other “non-sensory” factors that drive consumers’ liking in bread, for example, information about the product, its provenance/origin [18,19], salt content [20], perceived healthfulness, e.g., gluten-free status [21], as well as the type of flour [21,22,23]. Bread is a staple food across the world and is generally well liked with reported average hedonic scores of between 6.4 and 8.0 (using a 9-point hedonic scale). Freshness, flavour, and texture have been shown to impact liking among bread consumers [24,25].

European food exports and imports have grown by 20% and 18%, respectively, in the five years 2012–2017 [26]. Whilst some geographically protected products have become household names on a global scale, e.g., champagne and Parma ham, most products awarded Protected Geographical Indication(PGI), and Protected Designation of Origin(PDO) status are artisanal with small-scale production, and familiarity with the product tends to have a local focus. The key challenge when exporting such food products to markets with different cultural product expectations is to understand how the sensory perception of product-unfamiliar consumers maps to that of the product-familiar local population. This information enables the producer to develop sensory based marketing strategies using appropriate sensory language. This not only manages consumer expectation but improves the likelihood of commercial success [13,27,28].

Previous research using a trained sensory panel established a Quantitative Descriptive Analysis (QDA) profile for Waterford Blaa. Results have shown that Waterford Blaa has a unique sensory profile that distinguishes it from other white bread products, particularly by its characteristic thick flour dusting and strong toasted odour [29]. Furthermore, the sensory profile of Blaa differs between the three artisan bakeries producing it; Blaa A was deemed to have a sour odour, high amount of flour dusting appearance, and an intense, floury mouthcoating texture. Blaa B’s had a dense crumb structure appearance, strong oven baked flavour, and springy and firm texture, whereas Blaa C was characterised as having a strong oven baked odour and a thick exposed crumb appearance [29]. This study aims to examine whether cultural background/product familiarity, gender, and/or age impact consumers’ perceived liking of Waterford Blaa products and to determine whether it is possible to identify sensory characteristics of Waterford Blaa that influence liking, comparing product-familiar and product-unfamiliar consumer cohorts in Ireland and the UK, respectively.

## 2. Materials and Methods

### 2.1. Consumer Test

Two consumer tests were run with support from Sensory Dimensions Nottingham, one consumer study in Nottingham, United Kingdom (product-unfamiliar cohort) and the other consumer study in Waterford, Ireland (product-familiar cohort). All participants were recruited and screened using the following criteria: (a) over 18 years of age, (b) in good health, (c) no food related allergies, (d) UK only: consumption of white bread rolls at least once a week, (e) Ireland only: consumption of Blaa at least once a week), (f) signed written consent to take part in this study.

In the UK trial, n = 115 UK bread consumers (57 males; 68 females) were recruited from Sensory Dimensions volunteer database, while in the Irish study, n = 122 participants (54 males; 68 females) were recruited at The Waterford Food Festival in County Waterford, Ireland. Both studies were completed within one day, however, the Irish and the UK trials were undertaken on separate occasions 12 months apart. In each study, consumers participated in a 30 min tasting session in which they were asked to rate the overall liking of the products as well as their liking for each of the key sensory modalities (appearance, odour, flavour, texture). These questions were asked using a 9-point hedonic category scale that ranged from 1 (Dislike Extremely) to 9 (Like Extremely). In addition, consumers were asked to assess nine sensory attributes characteristic of Waterford Blaa: darkness of the crust; amount of flour dusting; sour and oven baked odour; salty, sour, and oven-baked flavours; firmness; and degree of mouthcoating. These were selected from descriptive sensory profiles of Blaa generated by a trained sensory panel during a previous research project [29]. Consumers used a 5-point Just About Right (JAR) category scale in which 1 represented “too little”, 3 represented “just about right”, and 5 represented “too much” for each of the selected characteristics. All subjects gave their informed consent for inclusion before they participated in the study. The study was conducted in accordance with the Declaration of Helsinki and GDPR regulations.

Because no novel ingredients were tested, specific ethical approval was not required. However, this study followed marketing and consumer research protocols, ISO 11136: 2017 Sensory analysis—Methodology—General guidance for conducting hedonic tests with consumers in a controlled area and IFST Professional Sensory Science Group guidelines.

### 2.2. Sample Preparation and Experimental Design

Waterford Blaa was produced by each of the three authorised artisanal producers using the ten stages outlined in Table 1. Blaa samples were produced and shipped by courier to arrive at the testing venue by the following morning for the consumer test. Each participant assessed Waterford Blaa products producers according to a balanced randomised experimental design. Each Waterford Blaa was presented sequentially to the consumers as a whole bread roll on a white paper plate with a plastic knife. For each question, participants were provided with instructions for assessment including the need to look at, smell, and bite into the Waterford Blaa for assessment of appearance, odour, flavour, and texture. Still water was used as a palate cleanser between samples, and participants were given a break of at least 5 min between samples to avoid sensory fatigue.

### 2.3. Data Analysis

Hedonic and JAR data from UK and Irish cohorts were analysed separately using XLSTAT Sensory (version 2018.5). 

Hedonic data for overall liking and liking for each modality were analysed using a two factor ANOVA model with Tukey’s multiple comparison test comparing the three Blaa products to determine if consumers differed significantly in their liking. Combined hedonic data from UK and Irish consumers were further analysed for each Blaa separately using a three factor ANOVA model to determine if age, gender, or culture impacted liking. For this analysis, data were split into three age categories: 18–34, 35–54, and >55 years old. 

JAR data were collapsed into three categories (too much, too little, and JAR), and the frequency of response (%) was calculated in each category. Penalty analysis was applied to UK and Irish data to determine the relative impact of specific product characteristics on overall liking.

## 3. Results and Discussion

Bread in its many forms is a world-wide staple. Typical overall liking scores for bread (generated using a 9-point hedonic scale) have been reported as 6.02 and 6.28 [15,30]. This study measured overall consumer liking for each modality as well as judgements about key sensory attributes to determine whether diversity exists between the perceptions of Irish consumers who are familiar with Waterford Blaa and UK consumer cohorts who are not. 

### 3.1. The Impact of Cultural Differences on Liking for Waterford Blaa

UK and Irish consumers differed in how much they liked the three Blaa products (Table 2). UK consumers liked Blaa C and B the most and Blaa A the least, whereas Irish consumers liked Blaa C and A the most and Blaa B the least. Liking for appearance, odour, texture, and flavour of the three Blaas followed the same trend as their results for overall liking.

UK consumers liked the appearance, the flavour, and the texture of Blaa C and B significantly more than Blaa A (*p* < 0.05) and the odour of Blaa C significantly more than Blaa A (*p* < 0.05); there were no significant differences in liking between Blaa C and Blaa B for any modality (*p* > 0.05). The mean scores for overall liking and each modality were very consistent for Blaa C (range: 6.6–6.7) and Blaa B (range: 6.2–6.4) and, therefore, it was not possible to determine the relative impact of each modality on overall liking for these two products. By contrast, UK consumers found appearance, flavour, and texture of Blaa A much less acceptable, particularly the appearance (mean score = 4.6), although they liked the odour (mean score = 6.0). It is probable that the sensory qualities affecting appearance and, to a lesser extent, flavour and texture, negatively impacted the result for overall liking.

Appearance attributes have been shown to be key in influencing liking and the decision to eat, as they shape a consumer’s first impression of a food product. For example, UK consumers liked the appearance of white bread significantly more (*p* < 0.0001) than red beetroot bread [19]. Norwegian consumers considered darker bread rolls to have more rye and therefore to be healthier than white bread, whilst Scottish, Estonian, and Czech consumers preferred the whitest bread [12]. In Ireland, a recent study showed 57% of the participants preferred white bread with males consuming more white bread than females [31]. In this study, Irish consumers liked the odour and the texture of Blaa C and A significantly more than Blaa B (*p* < 0.05) and the appearance and the flavour of Blaa C significantly more than Blaa B (*p* < 0.05); there was no significant difference in liking between Blaa C and Blaa A for any modality (*p* > 0.05). The mean scores for overall liking and each modality were >6 for Blaa C and Blaa A and, as with the UK study, it was not possible to determine the relative impact of each modality on overall liking. Irish consumers found appearance, flavour, and particularly texture (mean score = 5.2) of Blaa B less acceptable, and it is likely that these modalities negatively impacted overall liking. 

Whilst mean scores for the most liked Blaa C were similar for UK and Irish consumers, mean scores for the least liked Blaa A were markedly lower in the UK and below the 6.02 liking score previously reported [30]. Further analysis comparing data from both cohorts confirmed that Irish consumers liked Blaa A significantly more than UK consumers (*p* < 0.05). In this study, samples were presented blind with no additional information and, for UK consumers naïve to the product, a standard white floury bap would have been their most similar product experience. Irish consumers in Waterford were selected on the basis of having previously eaten Blaa. This meant that the Irish cohort was more familiar with the product and thus their expectations could be expected to follow the unique qualities of the Blaa product traditionally purchased within their family. It is likely, therefore, based on their prior knowledge and expectation, that UK and Irish consumers differed in their tolerance of and response to the unique qualities of Blaa A. This observation is consistent with other cross-cultural studies that found a positive correlation between product familiarity and product liking in Asian and Western panels—the rationale being that consumers who already knew a product reported feelings of safeness while, conversely, consumers not familiar with a product did not have this security, allowing the potential to give enhanced negative judgements when experiencing it for the first time [32].

Products displaying EU recognised PGI/PDO status enable the consumer to feel the product is safe and of superior quality. Awareness of PGI or PDO status among consumers has been linked to increased positive consumer association with the product and a willingness to pay more for products they perceive to be better and of higher quality [27,33,34]. Interestingly, results of the combined data follow the same trend as the UK results and do not mirror the preferences from the Irish cohort (Table 2). Whilst this is not surprising, as acceptability varied more among UK consumers, it highlights the importance of understanding consumer behaviour in each location rather than assuming that one location, or average results, can be used to predict liking in other populations. This is particularly important when marketing unique products from other countries/regions. For example, the unusual sensory qualities of Blaa A might have been more acceptable to UK consumers had they expected a difference and understood the cultural significance of those differences as opposed to treating the Waterford Blaa as just another bread roll. 

Comparison of UK and Irish results for each Blaa provided further evidence of cultural differences. Irish consumers liked Blaa A (*p* < 0.0001) significantly more than UK consumers, whereas UK consumers liked Blaa B (*p* < 0.05) significantly more than Irish consumers. Not only was Blaa C the most preferred by both consumer groups, there were no significant differences in liking between UK and Irish consumers (*p* > 0.05).

**Figure 1:**
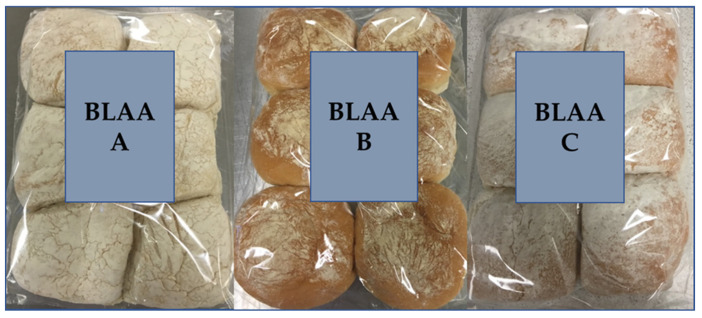
Appearance of the three Waterford Blaa products used in this study.

Whilst Blaa has PGI status and is manufactured to traditional recipes, the three Blaas used in this study had differing sensory profiles, in particular with regard to their appearance (Figure 1). Each Blaa’s unique sensory qualities were responsible for the significant differences in liking within each cohort and between UK and Irish consumers. Blaa A was the most polarising; whilst familiar to Irish consumers, its sensory characteristics were furthest away from those of a standard “floury bap” expected by UK consumers.

It is well established that perceived quality is not the same as expected quality [35]. Branding has a significant impact on consumer hedonic scores [36], and it has been shown that altering packaging information and other external cues can affect sensory perception [37] and influence consumer decisions [38]. Therefore, by positively marketing these differences to the UK consumer as the products’ unique selling point prior to launching such a product into the UK market, one could moderate the consumer expectation and consequently improve the sentiment towards the appearance of Blaas A and B.

### 3.2. The Impact of Age on Liking for Waterford Blaa

The population of developed countries is aging, and research into the differences in sensory perception between young and older consumers have found there to be considerable differences in rating of textural properties of food between the elderly and the young in a number of food products [39,40]. Factors impacting consumer liking have been shown to differ depending on age, younger consumers tending to value hedonics and price point when buying, whereas older consumers prioritise health benefits of a product [16,17,22]. 

In the context of Waterford Blaa, age had no impact on consumer liking within either Irish or UK consumers; there was no significant difference in overall liking nor liking for each modality between the three age categories (*p* > 0.05). There was also no significant interaction between age and Blaa for either the UK or Irish consumers.

### 3.3. The Impact of Gender on Liking for Waterford Blaa

Gender-stereotyped food associations exist in every culture [41], for example, in modern western culture, meat is often associated with men while salad is considered a more female associated dish [42]. A gender effect has been previously reported in consumer studies using orange juices and jam filled cakes [7], where males rated these products higher in likability than females.

In this study, the gender of Irish consumers did not impact liking for Waterford Blaa; there was no significant difference between males and females for overall liking nor liking for any modality (*p* > 0.05). For UK consumers, females liked the appearance of Blaa significantly less than males (*p* < 0.05). It is possible that UK females paid more attention to the appearance of the Blaas, or were more negatively impacted by its qualities, than UK males. This could be associated with females being more health conscious than men, as gluten products such as bread have been considered “not as healthy” as other carbohydrate sources [43].

### 3.4. Impact of Cultural Differences on Perception of Flour Associated Attributes

**Figure 2:**
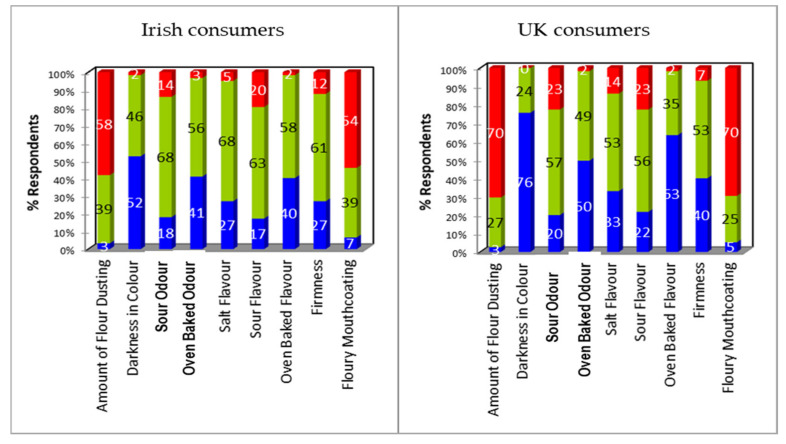
Deviation from ideal/just about right (JAR) for Blaa A from Irish and UK consumers. Data expressed as percent of responses from the collapsed JAR scale in which: Too little (blue) = rating of 1+2, Too much (red) = rating of 4+5, and Just right (green) = rating of 3.

Despite all three Blaas originating from Waterford and having PGI status, each Blaa had distinct qualities relating back to long-standing family recipes, particularly with respect to levels of flour coating. In this study, for example, anecdotal evidence suggested that Irish consumers easily identified the distinctive flour dusting on the top of Blaa A, the darker in colour of Blaa B, and the uniform shape of Blaa C (Figure 2).

Diagnostic assessment of the products using JAR scales highlighted differences in consumer judgements relating to the sensory attributes that characterise Blaa. Differences were not only evident when comparing between the products, they were also dependent on the geographic location of the consumer groups. Blaa A was considered to be the furthest from “just about right” for both UK and Irish consumers (Figure 2). Both cohorts judged the product to have too much flour dusting and therefore to be too pale; to have too little oven baked odour, salt, flavour, and oven baked flavour; to be too soft; and to have too much floury mouth-coating. Excessive flour dusting and mouth-coating as well as being too pale were the attributes that had the most impact with >50% of Irish and >70% of UK consumers expressing this view. It is possible that the excessive flour coating also reduced the intensity of salt and baked flavours. The judgements were more pronounced in UK consumers for whom a smaller proportion felt it was “just about right” for each sensory characteristic compared to Irish consumers.

**Figure 3:**
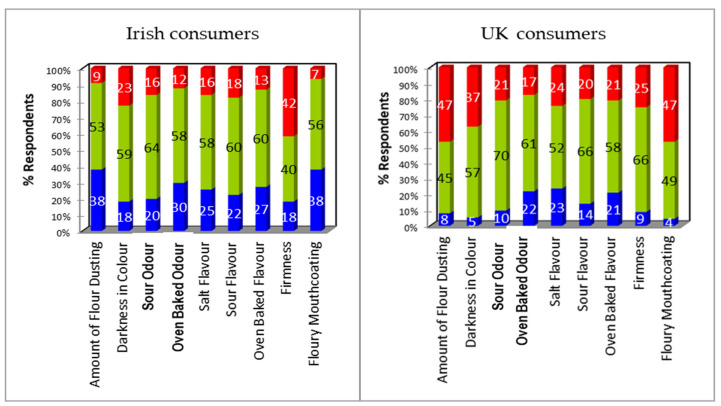
Deviation from ideal/just about right (JAR) for Blaa B from Irish and UK consumers. Data expressed as percent of responses from the collapsed JAR scale in which: Too little (blue) = rating of 1+2, Too much (red) = rating of 4+5, and Just right (green) = rating of 3.

Blaa B yielded different opinions from UK and Irish consumers (Figure 3). Judgements regarding flour dusting and mouthcoating were polarising; 47% of UK consumers felt there was too much, whereas 38% of Irish consumers felt there was too little. Irish consumers (42%) considered Blaa B to be too firm, whilst 37% of UK consumers felt that it was too dark. Results were similar for odour and flavour attributes.

**Figure 4:**
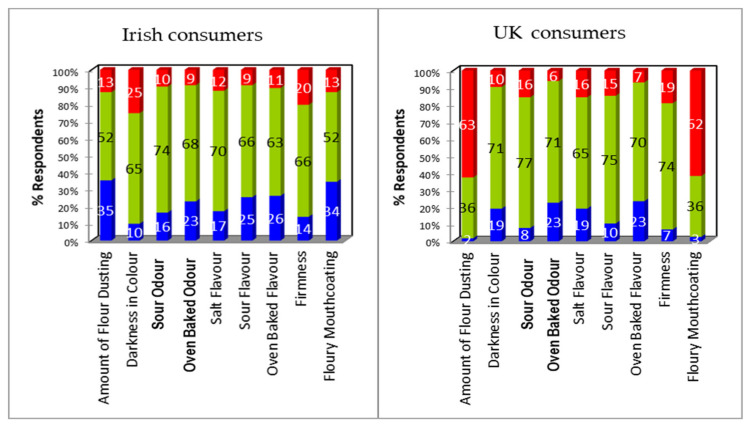
Deviation from ideal/just about right (JAR) for Blaa C from Irish (**left**) and UK (**right**) consumers. Data expressed as percent of responses from the collapsed JAR scale in which: Too little (blue) = rating of 1+2, Too much (red) = rating of 4+5, and Just right (green) = rating of 3.

Comparing the three products, Blaa C had the highest proportion of UK and Irish consumers who felt that it was “just about right” with regard to all odour and flavour attributes, darkness of colour, and firmness (Figure 4). Once again, consumers were less satisfied with the amount of flour dusting and mouthcoating and, consistent with Blaa B, their opinion was polarising; ~62% of UK consumers felt there was too much, whereas ~34% of Irish consumers felt there was too little.

Penalty analysis applied to JAR data identifies the relative impact of each attribute judgement on the degree of overall liking (Table 3). Weighted penalties >0.6 are indicative of attributes that drive disliking; they result from a high proportion of consumers sharing a judgement about a product, a large drop in overall liking because of that judgement (compared to those who felt the product was “just about right”), or a combination of both.

Based on weighted penalties, judgements related to flour dusting and mouthcoating, pale colour, and oven baked odour and flavour impacted the overall liking score for Blaa A in both UK and Irish consumers. Despite this, UK consumers liked Blaa A significantly (*p* < 0.0001) less than Irish consumers; this was due to two factors a) the mean liking score from Irish consumers who rated Blaa A “just about right” was significantly higher than the equivalent score from UK consumers and b) more Irish consumers judged Blaa A to be “just about right” than UK consumers. 

For UK consumers, it is probable that the significantly lower scores for appearance liking (excessive flour dusting and pale colour), flavour liking (weak baked flour flavour), and texture liking (soft texture and floury mouthcoating) were impacted by these attribute judgements. 

Whilst judgements regarding flour dusting and mouthcoating in Blaa B had been contrary between Irish and UK consumers, neither view translated into a notable penalty on overall liking. Irish consumers (>40%) considered Blaa B to be too hard, which resulted in its texture being liked significantly (*p* < 0.05) less than Blaa A and Blaa C and being their least liked product overall. UK consumers judged Blaa B to be too dark; however, despite a weighted penalty of 0.7, there was no obvious impact on the mean score for overall liking or appearance, mainly due to the high mean score from the 57% of consumers who judged it to be “just about right”. 

Blaa C was the most liked product for both Irish and UK consumers and had the highest proportion of consumers who felt that it was “just about right” with regard to all attributes except flour dusting and mouthcoating. There were no notable penalties from Irish consumers. Whilst 60% of UK consumers felt there was too much flour dusting and mouthcoating and did show a reasonable drop in overall liking, this was compensated by a particularly high overall liking mean score from those who judged it to be “just about right”.

A high proportion of UK consumers commented that all Blaas had too much flour dusting and mouth-coating, and it seemed the greater the flour dusting was, the more UK consumers disliked the product. The flour dusting and the characteristic floury mouthcoating are characteristic features of the product familiar to Irish Blaa consumers but a new experience for the UK consumers. The Irish consumers were all regular Waterford Blaa consumers who ate Blaa at least once a week, thus when asked at the end of the session whether Blaa A had too much flour -frequently occurring responses included “*Blaa is meant to be floury*” and “*Sure, you can knock off the excess flour on the plate if you don’t like it*”.

When considering export of local products into international markets heretofore unfamiliar with it, it is critical to understand consumer preferences. This can be used to obtain a direction for improving product performance [44] or to determine an appropriate marketing campaign to manage the market’s product expectation. This study achieved this by gathering information about consumers perception of the sensory characteristics of Waterford Blaa concurrently with overall liking scores [45] followed by analysing JAR and hedonic response data using penalty analysis [46]. JAR scales involve a direct comparison of the products with their conceptual “ideal”; by rating the perceived deviation from their ideal, they can deliver useful insights into product characterisation and market appeal [47]. However, this does presuppose that an ideal product exists and that the consumer knows what it is and can express what about the product is ideal/just about right. PGI/PDO producers entering a new market with unfamiliar consumers will gain useful insights into how their product compares with or differs from perceived similar products in that market, as the consumer ideal will perforce come from their eating experience.

## 4. Conclusions

This study aimed to investigate consumer acceptability for Waterford Blaa and determine if culture, age, and gender impacted liking among UK and Irish consumers. Familiarity with Blaa did impact consumer liking; this was particularly evident from the consumers’ responses to the heavy flour dusting, which is a unique property of Waterford Blaa. UK consumers felt that all Blaas had too much flour on top and too much of a floury mouthcoating. Consequently, Blaa A with the heaviest amount of flouring was the least preferred for UK consumers who liked it significantly less than Irish consumers (*p* < 0.05). Results for Blaa A and C suggest that flavour was also a key driver for UK consumers. Whilst Blaa C also had a heavily floured top, it delivered a stronger oven baked odour/flavour compared to Blaa A, which was enough to compensate for the flour coating and resulted in Blaa C being the most preferred by UK consumers. By contrast, whilst Irish consumers were aware of the same qualities in Blaa A and C, it did not negatively impact their results for overall liking or liking for any individual modality. Irish consumers were more impacted by the harder texture of Blaa B, which was their least preferred product. Age and gender did not impact liking for Blaas within Irish consumers, whereas for UK consumers, males liked the appearance significantly more than females.

Understanding how new markets might react to unfamiliar products and identifying the barriers to acceptability is important for the artisan bakers who may wish to expand into different markets. To this end, consumer education in a naïve market to raise consumer awareness and manage expectations is important, particularly for the more unusual product qualities, e.g., in the case of Waterford Blaa, the flour related attributes such as amount of flour dusting (appearance) and floury mouthcoating (texture). Results from this study offer useful feedback to the artisan bakers who could also take a second approach and slightly modify the production to match the consumer expectation. In the case of Blaa A, for example, a little less flour dusting and a slightly longer bake time could provide quick fixes to the pale “colour” and the too little “baking odour” and “baking flavour” attributes. Using sensory profiling, this consumer study underscores the importance of the traditional Research and Development guide to success, i.e., know your product; know your customer.

## Figures and Tables

**Table 1:** Stages of Waterford Blaa production.

Stage	Production Stage	Comment
**1**	Mixing	All ingredients mixed and divided into large pieces of dough
**2**	Resting	Dough is rested for 10–20 min
**3**	Pinning	Gives the dough characteristic round shape before baking
**4**	Sub-Dividing and resting (flour addition)	Dough subdivided into smaller pieces and rested and flour added
**5**	Flattening and rolling (Flour addition)	Dough flattened and placed on trays, rolled into prover for 1 h, more flour added and visual assessment
**6**	Proving (flour addition)	More flour added in one or two hand movements and proved
**7**	Baking	Dough baked in oven at 220–230 °C for 25–30 min
**8**	Assessment	Baker assesses dough before and after baking
**9**	Cooling	Blaa cooled for 1 h in ambient temperature
**10**	Packing	Blaa packaged into boxes or plastic wrapping

**Table 2:** Mean consumer liking of three Waterford Blaas for UK and Irish consumers. Data analysed using ANOVA with Tukey’s post hoc test.

	Overall Liking	Overall Liking		Appearance Liking		Odour Liking		Flavour Liking		Texture Liking	
BLAA	UK+IRISH	UK	IRISH	UK	IRISH	UK	IRISH	UK	IRISH	UK	IRISH
A	5.8 ^b^	5.2 ^b;B^	6.4 ^ab:A^	4.6 ^b;B^	6.5 ^ab;A^	6.0 ^b;B^	6.8 ^a;A^	5.2 ^b;B^	6.2 ^ab;A^	5.1 ^b;B^	6.1 ^a;A^
B	6.1 ^b^	6.3 ^a^	5.8 ^b^	6.3 ^a^	6.0 ^b^	6.3 ^ab^	6.3 ^b^	6.2 ^a^	5.7 ^b^	6.4 ^a;A^	5.2 ^b;B^
C	6.6 ^a^	6.6 ^a^	6.6 ^a^	6.7 ^a^	6.7 ^a^	6.7 ^a^	6.9 ^a^	6.7 ^a^	6.7 ^a^	6.7 ^a^	6.4 ^a^

Lowercase letters indicate significant differences from the Tukey’s post hoc between samples for each modality (*p* < 0.05). Uppercase letters indicate the significant differences between the cohorts for each Blaa within the one modality.

**Table 3:** Penalty analysis results for all attributes with a weighted penalty > 0.6 *.

Product	Location	Variable	JAR Group Mean Hedonic Score	Judgement	%	Mean Drop in Overall Liking	Weighted Penalty
Blaa A	Irish	Flour Dusting	7.3	Too much	58.2	1.7	1.0
		Darkness in Colour	7.7	Too little	52.5	2.5	1.3
		Oven Baked Odour	7.2	Too little	41.0	2.1	0.9
		Oven Baked Flavour	7.1	Too little	40.2	1.8	0.7
		Floury Mouthcoating	7.4	Too much	54.1	1.8	1.0
	UK	Flour Dusting	5.9	Too much	70.4	1.1	0.8
		Darkness in Colour	6.7	Too little	75.7	1.9	1.5
		Oven Baked Odour	5.8	Too little	49.6	1.3	0.6
		Oven Baked Flavour	6.2	Too little	63.5	1.5	1.0
		Firmness	6.1	Too little	40.0	2.0	0.8
		Floury Mouthcoating	6.4	Too much	69.6	1.7	1.1
Blaa B	Irish	Firmness	6.6	Too much	41.8	1.5	0.6
	UK	Darkness in Colour	7.1	Too much	37.4	1.9	0.7
Blaa C	UK	Flour Dusting	7.4	Too much	62.6	1.2	0.8
		Floury Mouthcoating	7.4	Too much	61.7	1.3	0.8

* JAR mean score = mean overall liking for those who rated “just about right”. Judgement = Too much or Too little from the collapsed scale. % = proportion of consumers expressing that judgement. Mean drop in overall liking = difference in overall liking between consumers with that judgement and those who rated “just about right”. Weighted penalty = % x Mean drop.
